# Reactive‐Oxygen‐Species‐Responsive Drug Delivery Systems: Promises and Challenges

**DOI:** 10.1002/advs.201600124

**Published:** 2016-06-08

**Authors:** Gurusamy Saravanakumar, Jihoon Kim, Won Jong Kim

**Affiliations:** ^1^ Center for Self‐Assembly and ComplexityInstitute for Basic Science (IBS)Pohang37673Republic of Korea; ^2^ Department of ChemistryPohang University of Science and Technology (POSTECH)Pohang37673Republic of Korea

**Keywords:** drug delivery, hydrogels, inorganic nanoparticles, polymeric nanoparticles, prodrugs, reactive oxygen species

## Abstract

Given the increasing evidence indicates that many pathological conditions are associated with elevated reactive oxygen species (ROS) levels, there have been growing research efforts focused on the development of ROS‐responsive carrier systems because of their promising potential to realize more specific diagnosis and effective therapy. By judicious utilization of ROS‐responsive functional moieties, a wide range of carrier systems has been designed for ROS‐mediated drug delivery. In this review article, insights into design principle and recent advances on the development of ROS‐responsive carrier systems for drug delivery applications are provided alongside discussion of their in vitro and in vivo evaluation. In particular, the discussions in this article will mainly focus on polymeric nanoparticles, hydrogels, inorganic nanoparticles, and activatable prodrugs that have been integrated with diverse ROS‐responsive moieties for spatiotemporally controlled release of drugs for effective therapy.

## 1 Introduction

Reactive oxygen species (ROS) are highly reactive ions and free radicals, including superoxide (O_2_^–^), hydroxyl radical (·OH), hypochlorite ion (OCl^–^), hydrogen peroxide (H_2_O_2_), singlet oxygen (^1^O_2_) and so on.^1^, ^2^ The major three sites of endogenous ROS generation are mitochondria, endoplasmic reticulum (ER), NADPH oxidase (NOX), which generate O_2_^–^.^2^ In addition, superoxide dismutases (SOD) and transition metals also participate in the generation of H_2_O_2_ and ·OH by reacting with O_2_^–^ and H_2_O_2_, respectively. The produced ROS play a vital role in physiological functions including the modulation of functions of proteins, production of several hormones, regulation of cell signaling, mediation of inflammation, and elimination of pathogens.^2^, ^3^, ^4^, ^5^ These versatile functions of ROS are modulated by its amount, duration and localization.^3^ In general, low levels of ROS regulate cell signaling pathway and promote cell proliferation, whereas high levels of ROS induce the non‐specific damage of proteins, lipids and DNA and kill the bacterial and pathogens.^2^, ^3^ Therefore, a lack of ROS or an excess of ROS can induce the several diseases including autoimmune disease, cardiovascular disease, neurodegenerative disease and so on.^3^, ^5^, ^6^, ^7^ In particular, the increase of ROS levels is at risk for mutating the cellular DNA, which is closely linked to the progression of several cancer cells.^2^, ^8^, ^9^, ^10^, ^11^, ^12^

**Figure 1:**
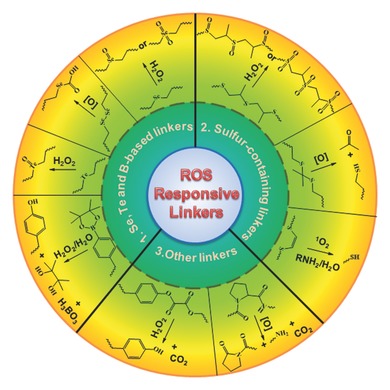
Representative ROS‐responsive linkers that have employed for the design of ROS‐responsive drug carriers.

This abnormal biochemical alteration in the disease sites has inspired researchers to exploit unbalanced ROS levels for developing target‐specific drug delivery systems.^1^ By utilizing ROS‐responsive materials and linkers (**Figure** **1**), various ROS‐responsive drug delivery systems have been developed and investigated for the therapeutic purposes. Sometimes, photosensitizers have been simultaneously utilized for synergistic therapeutic efficacy, which are one of the representative exogenous ROS sources and have ability to excite oxygen to its singlet state by using exogenous light energy.^13^, ^14^, ^15^

The aim of this article is to provide an overview of current research activities that center on reactive oxygen species (ROS)‐responsive drug delivery systems. We discuss comprehensively design principle and in vitro/in vivo evaluation of various ROS‐responsive drug delivery systems, including polymer‐based nanocarriers, hydrogels, inorganic nanoparticles‐based systems, and activatable prodrugs. Finally, we conclude this review article with future perspectives on this area.

## 2 ROS‐Triggered Drug Release from Polymeric Nanoparticles

Polymeric nanoparticles, formed through spontaneous self‐assembly of amphiphilic block copolymers, have been widely recognized as efficient carriers for delivery of wide range of imaging and therapeutic agents.^16^, ^17^, ^18^, ^19^ By controlling the hydrophilic‐lipophilic balance of block copolymers and the condition of solution state self‐assembly, we can easily tune their physical properties such as morphology and size which greatly influence in vivo biodistribution. Their core–shell nano‐sized structure enables polymeric nanoparticles to preferentially accumulate at the tumor site via enhanced permeation and retention (EPR) effect following systemic administration. It is also possible to engineer their surface with targeting ligands for more efficient intracellular delivery via receptor‐mediated endocytosis pathways. In addition to these targeting capabilities, integrating polymeric micelles with stimuli‐responsive units holds great promise because it allow us to fine tune release characteristics of the loaded drugs in a spatiotemporally controlled fashion at the target site. Though several stimuli‐responsive units that can respond to factors such as pH, temperature, light, enzyme or bioreducible environment have already been extensively explored in polymeric nanocarriers,^20^, ^21^, ^22^ the incorporation of ROS‐responsive units in polymeric nanoplatform for tunable drug release are only gaining attention very recently, as increasing evidence suggests that several pathogenic process are implicated with elevated ROS. In this section, we will systematically highlight the ROS‐responsive polymeric micelles that have been developed thus far. The discussions in the following sections are organized according to the types of ROS‐responsive units incorporated in the polymeric nanoparticles.

### 2.1 Organochalcogen (Se or Te) and Organoborane‐Based Linkers

#### 2.1.1 Selenium

Selenium is an essential trace element for animals and humans and a member of the chalcogen family (group 16) on the periodic table. It is also an important component of several vital enzymes such as glutathione peroxidase which plays significant role in protecting cells from oxidative damage. In particular, the lower bond energies of C‐Se (244 kJ mol^–1^) and Se‐Se (172 kJ mol^–1^) compared to the C‐S (272 kJ mol^–1^) and S‐S (240 kJ mol^–1^) make selenide or diselenide‐containing polymers more sensitive under mild stimuli conditions than the sulfur containing counterparts, which have inspired researchers to exploit the diselenide and monoselenide bond for developing stimuli‐responsive drug delivery systems.

**Figure 2:**
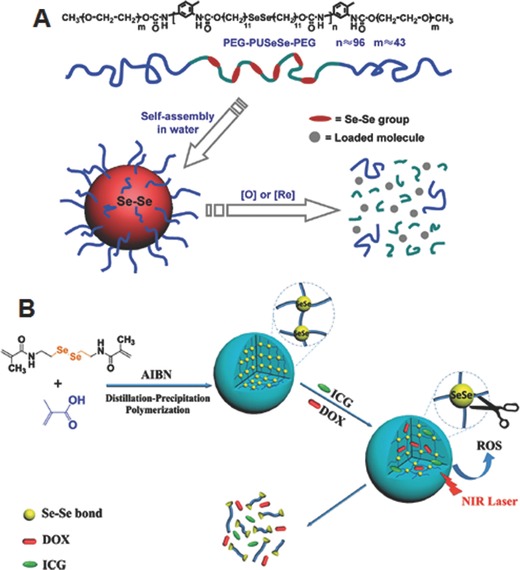
A) Chemical structure of PEG‐PUSeSe‐PEG and schematic illustrations of self‐assembled micelles and their redox responsive disassembly. Reproduced with permission.^23^ Copyright 2010, American Chemical Society. B) Illustration of preparation of diselenide‐crosslinked nanogels and their NIR‐regulated ROS‐activated degradation, following on‐demand controlled drug release. Reproduced with permission.^26^

Zhang and co‐workers first exploited the diselenide bonds for the development of ROS responsive micelles. They developed a triblock copolymer comprised of the poly(ethylene glycol)‐*b*‐polyurethane‐*b*‐poly(ethylene glycol) (PEG‐PUSeSe‐PEG) which contains diselenide bonds in the hydrophobic polyurethane block. The developed polymers could form the micelles via self‐assembly in water and facilitate the release of a model drug upon the glutathione (GSH) as well as H_2_O_2_ stimuli (**Figure** **2**A).^23^ In addition, the further research demonstrated that the micelles are also sensitive to even a low dose of γ‐radiation, such as 5 Gy, which is close to the radiation dose for patients during a single radiotherapy treatment.^24^ These results showed the potential applications in the combined radiotherapy and chemotherapy. The Xu groups developed light‐responsive drug delivery systems by adopting the ROS‐responsive behaviors of the PEG‐PUSeSe‐PEG micelles.^25^ In order to control the drug release on demand, they encapsulated porphyrins, one of the photosensitizers, in the core of PEG‐PUSeSe‐PEG micelles. The ^1^O_2_ produced from the photosensitizer under red light irradiation facilitated the cleavage of the diselenide bonds, which led to the disruption of the micelles and release of doxorubicin (DOX). In spite of the ability to control the drug release, the therapeutic potential of ROS‐responsive PEG‐PUSeSe‐PEG micelles still needs to be investigated for the practical applications. Similarly, Wang groups developed light‐responsive nanogel systems comprised of p(MAA‐Se‐Se‐MAA) by copolymerizing *N,N′*‐bis(methacryloyl) selenocystamine (BMASC) and metharylic acid (MAA) (Figure 2B).^26^ Indocyanine green (ICG) which has been approved by the US Food and Drug Administration (FDA) was employed to generate ^1^O_2_ for triggering the disassembly of diselenide‐crosslinked nanogels on demand. Upon near‐infrared (NIR) irradiation (785 nm), the enhanced DOX release and followed anticancer effects were demonstrated in vitro, which showed a potential in the use of targeted chemotherapy.

**Figure 3:**
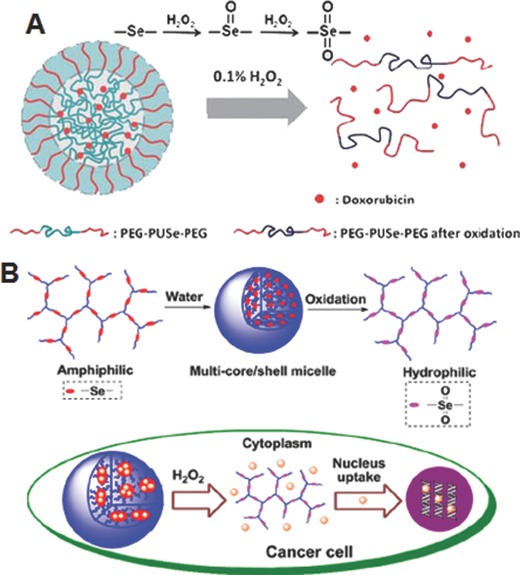
A) Schematic illustration of H_2_O_2_‐responsive dissassembly of Se‐containing block copolymer micelles. Reproduced with permission.^27^ Copyright 2010, Royal Society of Chemistry. B) Schematic illustration for preparation of self‐assembled nanoparticles from Se‐containing hyperbranched polymers and their intracellular drug release mediated by H_2_O_2_. Reproduced with permission.^29^ Copyright 2013, American Chemical Society.

The hydrophobic monoselenide can be converted to the relatively hydrophilic selenoxide or selenone under oxidative conditions. In order to examine the possibility of exploiting this unique characteristic of monoselenide for developing ROS‐responsive drug delivery systems, Zhang and co‐workers prepared the PEG‐PUSe‐PEG copolymer which is similar to the PEG‐PUSeSe‐PEG, but contains monoselenide in the hydrophobic PU block (**Figure** **3**A).^27^ PEG‐PUS‐PEG copolymer was prepared as a control, which contains monosulfide in the hydrophobic PU block instead of monoselenide. The developed micelles were exposed to the mild oxidative environment (0.1% H_2_O_2_ (v/v)) to investigate the ROS‐responsive structural disassembly. The thorough investigation including transmission electron microscope (TEM), Fourier transfer infrared (FT‐IR), X‐ray photoelectron spectroscopy (XPS) and nuclear magnetic resonance (NMR) demonstrated that the PEG‐PUSe‐PEG micelles are more sensitive to the oxidant conditions compared to the PEG‐PUS‐PEG micelles owing to the high activity of selenide groups to the oxidants. Zhang and co‐workers also developed amphiphilic block copolymers containing selenium in the side chain (PEO‐*b*‐PAA‐Se) by conjugating 11‐(benzylselanyl)undecan‐1‐ol to PAA of the poly(ethylene oxide‐*b*‐acrylic acid) (PEO‐*b*‐PAA).^28^ Similar to the PEG‐PUSe‐PEG, the developed micelles was disassembled under the mild oxidative environment. More interestingly, the addition of Vitamin C which is one of the mild reductants led to the re‐assembly of the micelles disassembled under ROS conditions, clearly indicating the high redox sensitivity of monoselenide group. This characteristic has inspired researchers to develop several ROS‐responsive drug delivery systems. Yan and co‐workers reported selenium‐containing (HBPSe) nanocarriers for the efficient DOX delivery (Figure 3B).^29^ The carriers were developed by biocompatible and biodegradable hyperbranched polymers composed of alternative phosphate segments and selenide groups in its backbone framework. The increased ROS levels of cancer cells facilitated the amphiphilic‐to‐hydrophilic transition of the polymers, which led to the rapid disassembly of the nanoparticles. As a result, loaded DOX was released, which showed the enhanced apoptosis of cancer cells at in vitro level. In particular, it is important to note that the polymers can be degraded by enzyme digestion after the drug release, which has raised the expectations in the applications of the ROS‐responsive drug delivery in practical cancer therapy.

#### 2.1.2 Tellurium

**Figure 4:**
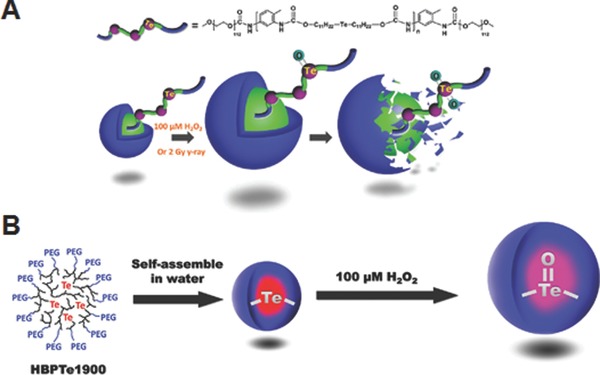
A) Chemical structure of linear amphiphilic tellurium‐containing copolymers, and morphological changes of the respective micelles triggered by H_2_O_2_ or 2 Gy γ‐ray radiation. Reproduced with permission.^30^ Copyright 2015, Royal Society of Chemistry. B) Preparation of self‐assembled micelles from Te‐containing hyperbranched polymers and their volume change by H_2_O_2_. Reproduced with permission.^31^ Copyright 2015, Royal Society of Chemistry.

Tellurium is another element of chalcogen family located below selenium in the periodic table. It is well known that electronegativity decreased as you go down a group, and therefore tellurium‐based polymers are expected to possess a higher sensitivity compared to that of selenium‐based ones. In spite of this important advantage, tellurium‐based polymers are still poorly explored for the design of ROS‐responsive drug carriers. Only in recent studies, Xu and co‐workers have synthesized two tellurium‐containing polymers with linear and branched architectures.^30^, ^31^ The linear Te‐containing amphiphilic copolymers (PEG‐PUTe‐PEG) self‐assembled into core–shell micelles in aqueous solution (**Figure** **4**A). These micelles showed sharp swelling and concomitant increase in particle size not only when treated to physiologically relevant concentration of ROS (100 μM H_2_O_2_), but also when exposed to clinically relevant dose of γ‐ray radiation (2 Gy) because ionizing radiation is known to generate ROS by water radiolysis.^30^ ^1^H NMR demonstrated that the swelling of micelles was mainly attributed to the disruption of the amphiphilicity via oxidation of tellurium on the hydrophobic polymeric backbone to the higher oxidation states. The sharp ROS responsivity of tellurium‐containing micelles under the above two conditions indicates that it might be a promising drug carrier for combination chemo and radio‐therapies. Similar to linear polymers, the hyperbranched tellurium‐containing polymers (HBPTe) also formed nano‐sized aggregates and displayed ROS‐responsive swelling (Figure 4B), which was greatly influenced by the degree of crosslinking of the polymer.^31^ Because of the ability to incorporate a larger number of tellurium into the hyperbranched three dimensional structures, these nanoaggregates are proposed to find potential use as scavenging materials to eliminate excess ROS in applications such as antioxidant therapy. Although both of the aforementioned systems showed morphological changes in the presence of ROS, their cytotoxicity or potential as ROS‐responsive drug carriers have not been studied in detail with model or therapeutic drugs.

It has been demonstrated in previous study that the specific coordination chemistry between tellurium and platinum could enable effective loading of platinum‐based anticancer drugs such as cisplatin and oxaliplatin into the micelles.^32^ Therefore, the strategy utilizing the coordination chemistry between tellurium and platinum would provide a novel route for developing ROS‐responsive drug delivery systems although it has not been investigated.

#### 2.1.3 Arylboronic Esters

In addition to their prominent function as important precursors in organic synthesis, the arylboronic acid or arylboronic ester functional groups have also been extensively employed in the design of functional materials, in particular as sensors because of their intrinsic binding affinities to diols, amino alcohols, cyanide and fluoride. The specific interaction between arylboronic acid and glucose has prompted the researchers to synthesis arylboronic acid‐based polymers and design glucose‐responsive polymer systems for controlled insulin delivery.^33^ Similarly, in recent years, the oxidation of arylboronic esters to phenols and boronic acid with H_2_O_2_ has also received great attention to explore polymers bearing arylboronic esters as drug carriers for oxidation‐responsive delivery.

**Figure 5:**
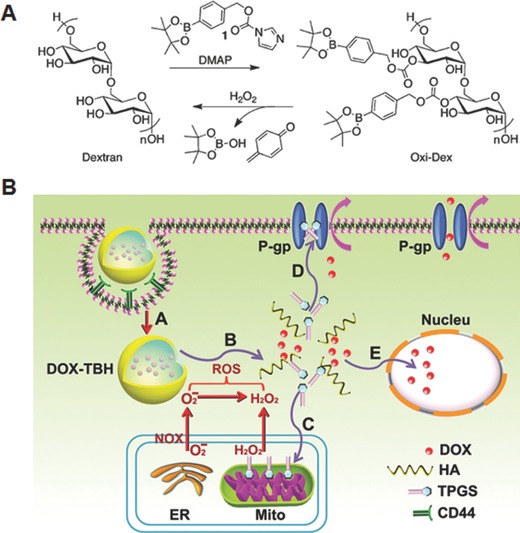
A) Synthesis and H_2_O_2_‐mediated degradation of Oxi‐DEX. Reproduced with permission.^34^ Copyright 2011, American Chemical Society. B) Schematic illustration of cellular uptake of regenerating anticancer nanosystem and proposed sub‐cellular drug release. A: CD44‐mediated endocytosis, B: cleavage of arylboronate ester by ROS triggered disassembly and release of DOX and TPGS, C: upon its interaction with mitochondrial respiratory complex II, TPGS induced ROS generation for complete disassembly of nanosystem, D: TPGS acts as an effective P‐gp inhibitor to hinder the efflux of DOX, E: DOX and ROS induced cell death in nucleus. Reproduced with permission.^35^ Copyright 2014, Elsevier.

An early effort in the utilization of arylboronic ester‐based polymers as ROS‐responsive drug delivery vehicles was made by Frechet and co‐workers.^34^ For this, they synthesized an oxidation‐sensitive dextran (Oxi‐DEX) derivative by conjugating hydroxyl groups of dextran with imidazoyl carbamate‐activated phenyl boronic ester (**Figure** **5**A). By employing this Oxi‐Dex derivative, H_2_O_2_‐responsive nanoparticles with size in the range of 100–200 nm were prepared using standard microemulsion method. Because of the solubility switch resulting from the oxidation of arylboronic esters, these nanoparticles were quickly disassembled in phosphate buffer containing 1 mm H_2_O_2_, but they were stable in the absence of H_2_O_2_. As these nanoparticles were fabricated using biocompatible dextran as major constituent, they were nontoxic to both HeLa human cervical epithelial cells and RAW 264.7 murine macrophages. The potential of these nanoparticles as intracellular antigen delivery system for vaccine applications was investigated by loading chicken egg albumin (OVA). Interestingly, OVA‐loaded Oxi‐DEX nanoparticles showed significantly higher (by 27 fold) MHC I presentation as compared to the OVA‐loaded oxidation‐insensitive conventional polymeric nanoparticles, indicating their high potential as ROS‐responsive carriers for the delivery of hydrophilic biomacromolecular drugs. Zhang and co‐workers developed another interesting ROS‐triggered and regenerating anticancer nanosystem using an amphiphilic polymer (TBH), which was synthesized by conjugating D‐α‐tocopherol polyethylene glycol succinate (TPGS) to hyaluronic acid backbone via arylboronic ester linkage.^35^ The amphiphilic conjugate formed self‐assembled nanoparticles, in which anticancer drug DOX was effectively loaded by simple dialysis method. The DOX‐loaded nanoparticles showed tumor cells‐specific uptake in vitro via CD44‐medidated endocytosis and disassembled in the intracellular ROS environment through cleavage of arylbronic acid ester bond, which enabled to trigger the release TPGS and DOX (Figure 5B). The interaction of released TPGS with mitochondrial respiratory complex II induced further generation of ROS, which ensures complete dissociation of nanoparticles. Owing to P‐glycoprotein (P‐gp) inhibitory mechanism of TPG and high level of intracellular DOX concentration, the nanoparticles showed enhanced cytotoxicity in MCF‐7/ADR cells. The nanoparticles demonstrated prolong in vivo circulation in the blood and enhanced tumor targeting characteristics by combination of passive and active targeting mechanism. Further the nanoparticles showed excellent antitumor efficacy and a high survival rate in vivo as compared to free DOX and the control nanoparticles without arylboronic ester linkage.

**Figure 6:**
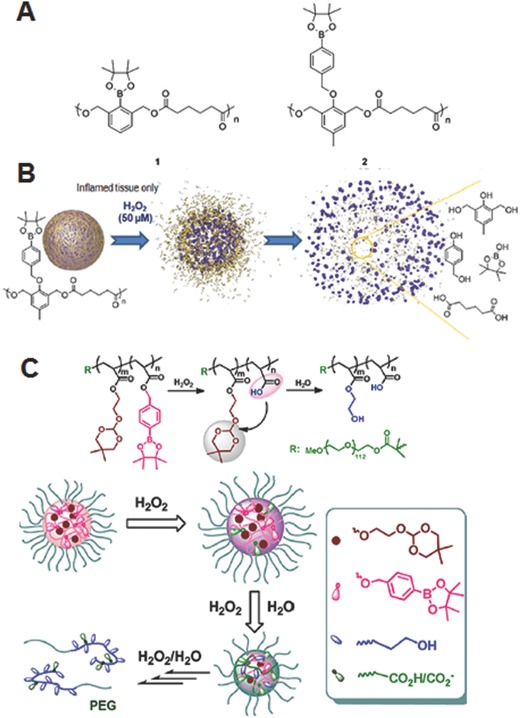
A) Chemical structures of polymers bearing arylboronic ester group linked directly (1) or via an ether group (2). B) Illustration of H_2_O_2_ mediated disassembly of nanoparticles formed using (2) and their degradation products. Reproduced with permission.^36^ C) Illustration of disassembly of oxidation and pH‐dual responsive micelles bearing pendent arylboronic ester and ortho ester groups and their mechanism of disassociation (oxidation accelerated hydrolysis of ortho ester). Reproduced with permission.^40^ Copyright 2013, American Chemical Society.

In addition to the arylboronic ester‐modified polysaccharide systems discussed above, synthetic copolymers incorporated with arylboronic ester units at the main backbone or side chains have also been synthesized and investigated as ROS‐responsive drug carriers. For example, Almutairi and co‐workers reported H_2_O_2_‐responsive nanoparticles using two complementary polymers bearing arylboronic ester groups, either linked directly or via an ether linkage to the main backbone (**Figure** **6**A).^36^ The nanoparticles were formulated via an oil/water emulsion technique. Upon exposed to physiologically relevant concentration of H_2_O_2_, the nanoparticles prepared using the polymer with an ether linkage showed much faster degradation compared to the other one (Figure 6B). Notably, these nanoparticles were completely degraded in activated neutrophils with high levels of ROS, and showed 2‐fold enhancement of model drug release compared to the control poly(lactic‐*co*‐glycolic acid) (PLGA) nanoparticles.

One of the problems associated with polymers bearing arylboronic esters is the generation of highly reactive quinone methide (QM) intermediates upon degradation. Under in vivo conditions, these QMs can react with biomomolecules including proteins and DNA, and induce harmful side effects. Further, QMs are also capable of depleting glutathione, a key intracellular antioxidant, leading to an altered redox balance within the cells. To address this issue, Li and co‐workers designed nanoparticles using a new type of amphiphilic poly(amino ester)s containing pendant arylboronic ester units.^37^ Upon H_2_O_2_‐triggered degradation of the nanoparticles, the built‐in amino groups effectively quenched the generated QMs, thereby alleviating the biocompatibility concerns of the degradation products. Similarly, efforts have also been made to improve the polymer biocompatibility by constructing the arylboronic ester‐based nanoplatforms using β‐cyclodextrin (β‐CD) which exhibits excellent biocompatibility and low immunogenicity as major a constituent of the carrier.^38^

Because of the complex biological environment, in recent years, polymeric nanoparticles that have been integrated with multiple stimuli‐sensitive units are being widely investigated because such systems enable to achieve desirable on demand drug release kinetics. In this regard, nanoparticles bearing an additional stimuli‐sensitive unit along with ROS triggers have also been designed. For example Jia and co‐workers synthesized two azomethine‐based oligomers bearing arylboronate ester and imine moieties, as ROS‐ and pH‐responsive units respectively, in the main chain through a condensation polymerization from a dialdehyde monomer containing a boronate group and an aromatic amine or an aliphatic amine.^39^ The nanoparticles fabricated using these oligomers were degraded either by oxidative conditions (H_2_O_2_) or acidic environment (low pH), leading to the release of payload in a controlled manner. In particular, the nanoparticles formed using oligomers with aliphatic amine showed a faster degradation rate in the presence of H_2_O_2_, especially in a weak acidic environment, as compared to the one made using aromatic amine. In another study, Li and co‐workers synthesized interesting oxidant/pH dual‐responsive micelles using an amphiphilic block copolymers bearing pendant arylboronic ester and ortho ester groups (Figure 6C).^40^ Upon oxidation by H_2_O_2_, the arylboronic ester group within the micellar core rapidly oxidized to produce carboxylic acid, which subsequently improved the polarity of local microenvironment of hydrophobic core and facilitated the uptake of water molecules, and accelerated the hydrolysis of ortho ester group. The degradation kinetics of the micelles was controlled by changing the polymer composition, concentration of H_2_O_2_ and pH condition. Notably, some of the micelles were extremely sensitive to the biorelevant concentration of H_2_O_2_ (50 μm). Considering the acidic conditions of tumor extracellular matrix and intracellular compartments of the cells such as endosomes and lysosomes, these ROS and pH‐dual responsive carriers might have great potential for precise controlled release of anticancer therapeutics.

### 2.2 Sulfur‐Containing Linkers

#### 2.2.1 Thioether

**Figure 7:**
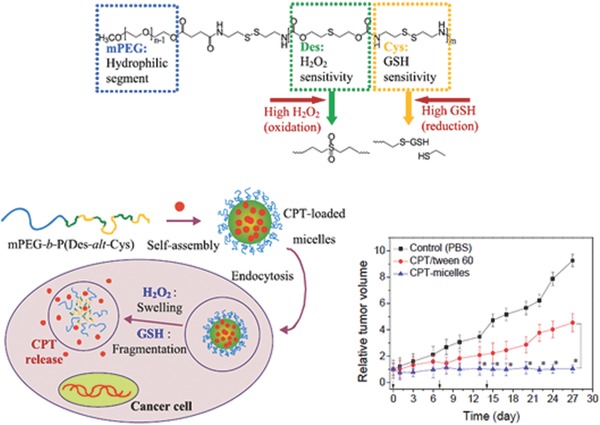
Chemical structure of oxidation and reduction‐responsive block copolymer, and intracellular CPT release triggered by ROS and glutathione. Under high levels of H_2_O_2_ in cancer cells, the thioether linkers undergo hydrophobic‐to‐hydrophilic transition, which induces the swelling of the micelles. At the same time, the intracellular redox conditions trigger the clevage of disulfide bonds. These dual‐responsive behaviors deformed the micelles, which induces the drug release. The graph represents in vivo tumor growth inhibition of CPT‐loaded micelles. Reproduced with permission.^44^ Copyright 2015, Elsevier.

As discussed above,^27^ thioether group is similar to the monoselenide group, which undergoes a phase transition from hydrophobic to hydrophilic under ROS conditions. Walker's group explored the potential of thioether group in the development of ROS‐responsive drug delivery systems by preparing the nanoparticles composed of poly(propylene sulfide) (PPS) containing thioether in its backbone.^41^ They demonstrated that the oxidation of thioether in the PPS by the hypochlorite led to the swelling of the nanoparticles. In particular, the ROS‐generating enzymes such as chloroperoxidase (CPO) and human myeloperoxidase (hMPO) which generate HOCl at acidic pH induced the increased release of a model drug, which showed the practical potential of the thioether‐mediated drug delivery systems. Li groups reported the oxidation‐responsive drug delivery system by adopting the poly _L_‐cystein derivatives (poly(_L_‐EG*_x_*MA‐C)*_n_*) containing thioethers in polypeptide side chains, which undergoes interesting structure transition under H_2_O_2_ conditions.^42^ They found that the oxidation of thioether triggered the conformation change of poly(_L_‐EG*_x_*MA‐C)*_n_* from β‐sheet to random coil. The increase of solubility as well as the structural changes have inspired the researchers to exploit poly(_L_‐EG*_x_*MA‐C)*_n_* as a hydrophobic block of the micellar systems. As a result, PEG_45_‐*b*‐poly(_L_‐EG_2_MA‐C)_22_ micelle was prepared and showed the H_2_O_2_‐responsive DOX release. These results have led to several researches to investigate the therapeutic potential of the delivery systems utilizing thioether linkers. Zhu and co‐workers developed H_2_O_2_‐responsive hyperbranced polymeric micelles for the delivery of 7‐ethyl‐10‐hydroxy‐camptothecin (SN38) and cinnamaldehyde (CA).^43^ SN38 was conjugated to the hydrophilic hyperbranced polyglycerol (HPG) via ROS‐responsive thioether linker and the CA which induces apoptotic cell death via ROS production was encapsulated in the resultant SN38‐conjugated HPG micelles. The H_2_O_2_‐responsive disassembly as well as drug release were demonstrated, which led to the efficient cytotoxicity on the several cancer cells including MCF‐7, HeLa and HN‐4. More importantly, the co‐delivery of SN38 and CA exhibited improved cell apoptosis compared to the single drug administration, which shows great potential of the strategy utilizing ROS‐responsive linkers, ROS generating drugs and chemotherapeutic drugs. Recently, Lo et al. evaluated the potential of thioether‐based ROS‐responsive drug delivery systems at in vivo level (**Figure** **7**).^44^ They developed a dual redox responsive polymer composed of hydrophilic methoxy PEG and hydrophobic block response to H_2_O_2_ and GSH. The thioether and disulfide bonds were employed to impart the oxidation‐ and reduction‐responsiveness, respectively. The micelles containing camptothecin (CPT) exhibited oxidation‐ and reduction‐responsiveness and interestingly showed the cell‐dependent anticancer effects. In particular, the micelles induced efficient anticancer effects on the cancer cells compared to the free CPT although they showed the negligible cytotoxicity to the normal tissue cells which have low levels of ROS and GSH. Based on these results, the therapeutic potential of the dual responsive CPT delivery micelles was evaluated in HCT116 tumor‐bearing mice. The micelles exhibited improved in vivo antitumor efficacy compared to the free drug, which open the possibility of the practical applications of thioether‐bearing drug delivery systems.

#### 2.2.2 Thioketal

Thioketals are commonly used as carbonyl protecting group in organic synthesis because of their known stability to acidic and basic conditions. However, thioketals can be cleaved through oxidative means. By taking advantage of this chemistry, Murthy and co‐workers developed thioketal nanoparticles for oral delivery of siRNA to the inflamed intestinal tissues, which exhibit abnormally high levels of ROS.^45^ The siRNA‐loaded nanoparticles were prepared by complexing siRNA with a cationic lipid (1,2‐dioleoyl‐3‐trimethylammonium‐propane) followed by loading the resulting complexes into nanoparticles composed of ROS‐responsive thioketal linkages incorporated polymer, poly(1,4‐phenyleneacetonedimethylene thioketal) (PPADT). PPADT was readily synthesized using 1,4‐benzendimethanethiol and 2,2‐dimethoxypropane via a step‐growth polymerization method. As anticipated, these thioketal nanoparticles protected the complexes degradation in the harsh gastrointestinal tract fluids, but effectively unpacked the loaded siRNA at the sites of intestinal inflammation via ROS‐mediated polymer degradation. Based on these results, PPADT nanoparticles were investigated as a carrier for small molecule anticancer drug paclitaxel (PTX).^46^ Compared to placebo PPADT nanoparticles, PTX‐loaded PPADT nanoparticles demonstrated significantly high cytotoxicity in PC‐3 prostate cancer cells which exhibit elevated levels of ROS compared to other cancer cells. This study demonstrated the feasibility of the development of targeted drug delivery using the ROS‐responsive thioketal‐based polymers. It should also be emphasized that thioketal‐based ROS‐responsive polymeric carriers are not widely explored as compared to others.

#### 2.2.3 Vinyldithioether

**Figure 8:**
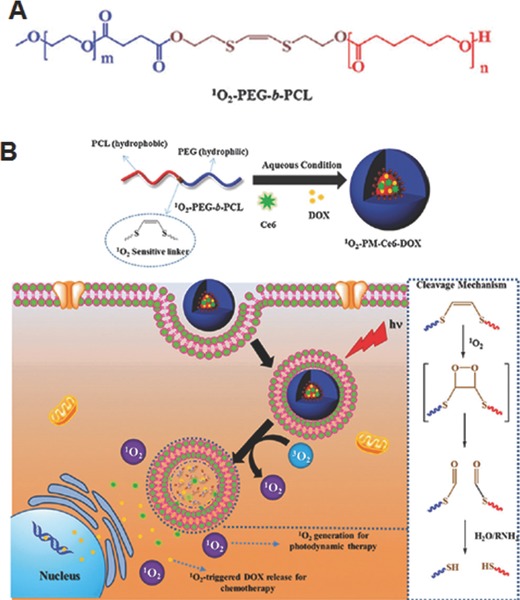
A) Chemical structure and B) Illustration of preparation of Ce6 and DOX co‐loaded micelles and cellular uptake and visible light triggered ^1^O_2_‐mediated intracellular co‐delivery of DOX and Ce6 for combination chemo and photodynamic therapy. Reproduced with permission.^48^ Copyrigh 2015, Royal Society of Chemistry.

Among various reactive oxygen species, ^1^O_2_ undergoes [2+2] cycloaddition with electron rich olefins such as vinyldithioether to form unstable dioxetane intermediates, which spontaneously fragment to generate two carbonyl products. Based on this chemistry, Baugh et al. synthesized cleavable β‐CD dimer as carrier of photosensitizer zinc phthalocyanine (ZnPc) for photodynamic therapy (PDT).^47^ More recently, our group developed polymeric micelles based on biocompatible poly(ethylene glocol)‐*b*‐poly(caprolactone) (PEG‐*b*‐PCL) copolymer bearing a ^1^O_2_‐sensitive vinyldithioether linker at the core–shell junction for effective intracellular co‐delivery of photosensitizer, chlorine e6 (Ce6), and anticancer drug, DOX (**Figure** **8**).^48^ Ce6 and DOX co‐loaded micelles were prepared using simple dialysis method. *In vitro* cell experiments using PC3 human prostate cell line revealed that these co‐loaded micelles under visible light irradiation more effectively released DOX inside the cells, and showed a higher antitumor efficacy as compared to control micelles without the linker. This enhanced therapeutic efficacy was mainly attributed to the generation of ^1^O_2_ and the light‐induced release of DOX upon ^1^O_2_‐mediated cleavage of the vinyldithioether linker of the micelles. As light is an integral part of PDT, these micelles might have great potential for photodyamically triggered drug release for effective combined chemotherapy and PDT.

### 2.3 Other Linkers and Strategies

#### 2.3.1 Aryloxalate

It has been well known that aryloxalates can easily react with oxidant H_2_O_2_ to form 1,2‐dioxetanedione, which rapidly decompose into carbon dioxide. Thus, strategically placing aryloxalate ester bond within the polymeric nanoparticles can induce their degradation and release the cargo upon exposing to H_2_O_2_.

**Figure 9:**
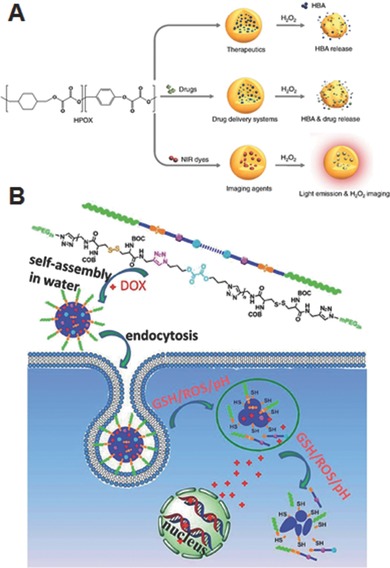
A) H_2_O_2_‐activatable HPOX nanoparticles as imaging agents, therapeutics, and site‐directed drug delivery systems for ischemia‐reperfusion injury. Reproduced with permission.^50^ Copyright 2013, Elsevier. B) Preparation of DOX‐loaded PRDSP nanoparticles, their uptake and intracellular microenvironment triggered release of DOX. Reproduced with permission.^53^ Copyright 2015, Royal Society of Chemistry.

Kang and co‐workers developed H_2_O_2_‐responsive *p*‐hydroxybenzyl alcohol (HBA)‐incorporated polyoxalate copolymer (HPOX) nanoparticles with strong antioxidant properties for therapeutic and diagnostic purposes (**Figure** **9**A).^49^, ^50^ HBA is a major active pharmaceutical ingredient in Gastrodia elata, which has been widely used as herbal agents for the treatment of oxidative stress‐related diseases. The copolymer was synthesized by one‐pot condensation reaction between oxalyl chloride, 1,4‐cyclohexanedimethanol and HBA. The HPOX nanoparticles were formulated by an oil‐in‐water emulsion method. The labile oxalate ester bonds on the copolymer can undergo complete hydrolytic or H_2_O_2_‐mediated degradation, which enables to release the active form of HBA to exert its therapeutic effect. *In vitro* evaluation of HPOX nanoparticles using LPS‐activated RAW 264.7 cells demonstrated strong antioxidant and anti‐inflammatory activities by inhibiting the production of nitric oxide and reducing TNF‐α.^49^ The HPOX nanoparticles were also evaluated for H_2_O_2_ imaging and as targeted drug delivery vehicles for the treatment of ischemia‐reperfusion injury.^50^ In another study, a similar type H_2_O_2_‐responsive antioxidant nanoparticle was fabricated by substituting the HBA with vanillyl alcohol (VA), another antioxidant. This polyoxalate copolymer bearing VA (PVAX) nanoparticle exhibited significant antioxidant, anti‐inflammatory and anti‐apoptotic activities in both in vitro and in vivo models of ischemic/reperfusion injury.^51^ Interestingly, PVAX nanoparticles prevented DOX‐induced cardiac dysfunction.^52^ Taken the advantages of excellent biodegradability, antioxidant property, and low cytotoxicity, these HPOX and PVAX nanoparticles have great potential as therapeutics for oxidative stress‐associated disease.

To increase the sensitivity of the peroxalate‐based nanoparticles, Wu et al. synthesized a dual sensitive ABA‐type amphiphilic triblock polymer (PRDSP), wherein the middle hydrophobic block was incorporated with peroxalate ester units and disulfide bonds repeatedly, and PEGs were selected as outer hydrophilic blocks.^53^ The DOX‐loaded PRDSP nanoparticles showed accelerated release of DOX under biologically relevant concentration of glutathione (10 mm) and H_2_O_2_ (10 mm) compared to the physiological conditions (Figure 9B). Confocal laser scanning microscopy and flow cytometric analysis showed effective delivery of DOX into the cytoplasm and nucleus of cells.

#### 2.3.2 Ferrocene

Because of their unique redox properties, ferrocene‐containing polymers are highly promising as a scaffold material for designing drug carriers, among several other applications.^54^, ^55^, ^56^ As the ferrocene unit can be converted from its hydrophobic neutral state to the hydrophilic charged species ferrocenium cation upon oxidation, the resulting hydrophobic‐to‐hydrophilic transition can be utilized to trigger ROS‐mediated drug release. For example, Zhang and co‐workers synthesized ferrocene‐containing amphiphilic block copolymers poly(ethylene oxide)‐*b*‐poly(2‐formal‐4‐vinylphenyl ferrocenecarboxylate) (PEG‐*b*‐PMAEFc) via atom transfer radical polymerization.^57^ In aqueous solution, this copolymer formed self‐assembled vesicular nanostructures, but their morphologies can be effectively tuned by the factors such as polymer concentration, composition or addition of β‐CD which is an appropriate host molecule that can form 1:1 inclusion complex with ferrocene unit. The detailed investigation revealed that increasing the amount of β‐CD induces a variety of intriguing structures, such as interconnected aggregates, compacted microstructures and large compound vesicles. The Rhodamine B (RB)‐loaded polymeric vesicles showed an accelerated release of RB in the presence of 1.4% H_2_O_2_, as compared to that one without any oxidant. It is also worth to mention here that reversible host‐guest interactions between β‐CD and ferrocene have also been exploited to construct voltage‐responsive supramolecular vesicles for drug delivery applications.^55^ Various ROS‐responsive polymeric nanoparticles are summarized in **Table** **1**. In general, these nanoparticles showed disassembly and concomitant drug release upon exposing to ROS, either through degradation of oxidation‐labile linkers or hydrophilic‐to‐hydrophobic transition.

**Table 1:** Polymeric nanoparticles that have been developed for ROS‐triggered drug release

Linkage	Polymer	Active agents or Model drug	ROS‐trigger	Cell line	References
Diselenide	PEG‐PUSeSe‐PEG	RB	H_2_O_2_ (0.1–0.01%) or GSH (0.1–0.01 mg mL^–1^)	‐	23
		DOX	γ‐radiation (5 Gy)	HepG2	24
	P(MAA‐SeSe‐MAA)	DOX and ICG	Light (600–780 nm)‐induced ROS	HeLa	26
Monoselenide	PEG‐PUSe‐PEG	DOX	H_2_O_2_ (0.1%)	‐	27
	PEO‐*b*‐PAA‐Se	NR	H_2_O_2_ (0.1%)	‐	28
	HBPSe	DOX	H_2_O_2_ (0.1 mm)	HeLa and NIH‐3T3	29
Telluride	PEG‐PUTe‐PEG	‐	H_2_O_2_ (100 μm) γ‐radiation (2 Gy)	‐	30
	HBPTe	‐	H_2_O_2_ (100 μm)	‐	31
Arylboronic ester	Oxi‐Dex	OVA	H_2_O_2_ (1 mm)	HeLa and RAW 264.7	34
	TBH	DOX	H_2_O_2_ (1 mm)	MCF‐7/ADR	35
	P1 and P2	NR and FD	H_2_O_2_ (50–100 μm)	Raw264.7 and dMPRO	36
	Azomethine‐based oligomer	NR	H_2_O_2_ (10 mm) and/or H^+^	HeLa	39
	Ortho‐ester containing block copolymer	NR	H_2_O_2_ (50 μm)	‐	40
Thioether	PPS	NR and Reichardts dye	NaOCl (25 ppm), CPO and hMPO with NaCl (200 mm) and H_2_O_2_ (500 μm)	‐	41
	Poly(L‐EG*_x_*‐MA‐C)*_n_*	DOX	H_2_O_2_ (0.3%)	‐	42
	HPG‐2S‐SN38	CA	H_2_O_2_ (1–5 mm)	HeLa, MCF‐7 and HN‐4	43
	mPEG‐*b*‐P(Des‐alt‐Cys)	CPT	H_2_O_2_ (1–100 μm) and GSH (1 μm–20 mm)	HCT116 and L929	44
Thioketal	PPAD	TNF‐α‐siRNA	KO_2_	RAW 264.7	45
		PTX	KO_2_ (10 mm) or H_2_O_2_ (10 mm)	PC‐3	46
Vinyldithioether	β‐CD	ZnPC	Light‐induced ROS	‐	47
	PEG‐*b*‐PCL	Ce6 and DOX	Light (660 nm)‐induced ROS	PC‐3	48
Aryloxalate	HPOX	HBA, 4‐AN	H_2_O_2_ (100 μm)	RAW 264.7	49
	PVAX	VA	H_2_O_2_ (0.1 mm)	C2C12	51
	PRDSP	DOX	H_2_O_2_ (10 mm) or GSH (10 mm)	L929, A549 and HeLa	53
Ferrocene	PEG‐*b*‐PMAEFc	RB	H_2_O_2_ (1.4%)	‐	57

2.3.3 Other Strategies

**Figure 10:**
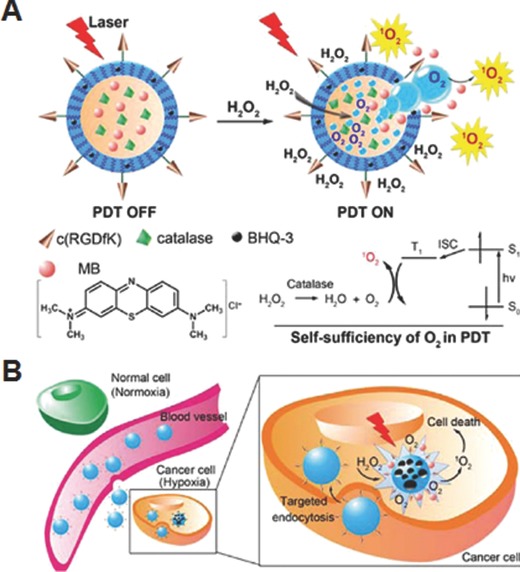
A). Schematic illustration of mechanism of H_2_O_2_‐triggered release of photosensitizer and O_2_ to accomplish PDT. B) Targeted intracellular generation of O_2_ and ^1^O_2_ by the nanoparticles for efficient PDT against hypoxic tumor cell. Reproduced with permission.^60^ Copyright 2015, American Chemical Society.

Gas‐generating polymer nanoparticles are emerging as a potential carrier system for targeted triggered drug release.^58^, ^59^ In this system, the increase in internal pressure as a result of gas generated from the nanoparticles induces the disassembly of nanostructures and release of the loaded drugs. Thus, it does not necessarily demand the incorporation of stimuli‐responsive linkages within the polymeric nanostructures for drug release. The targeted triggered drug release from this system is accomplished by stimuli‐controlled gas generation in response to specific microenvironmental changes associated with the diseases. By taking the advantages of O_2_‐generation from the reaction between catalase and H_2_O_2_, Guo and co‐workers prepared an interesting carrier system that can generate O_2_ and deliver anticancer drugs in response to high H_2_O_2_ levels in the tumor microenvironment.^59^ Biocompatible PLGA nanoparticles were chosen as the drug carrier, in which catalase and platinum‐based drug as O_2_‐generating agent and anticancer agent respectively, were loaded into aqueous core by double emulsion method. When these nanoparticles are taken up by the tumor cells, the O_2_ generated by the catalase from intracellular H_2_O_2_ not only facilitates drug release but also be helpful in overcoming hypoxia‐induced MDR which limits the effectiveness of chemotherapy. Thus, the co‐loaded nanoparticles demonstrated improved therapeutic efficacy against cisplatin resistant cell lines that are often appear to be in hypoxia. In a subsequent study, the authors investigated the potential of O_2_‐generating nanoparticles for PDT against hypoxic cells (**Figure** **10**A).^60^ Although PDT that produces cytotoxic ^1^O_2_ by excitation of photosensitizer with light of appropriate wavelength in the presence of oxygen has become promising treatment modality for several forms of cancers, the depletion of oxygen by photochemical consumption during PDT process and inherent tumor hypoxia of solid tumor significantly limit their successful clinical applications. Owing to O_2_‐generating capacity at hypoxic conditions, it has been assumed that O_2_‐generating nanoparticles might hold great promise to overcome tumor hypoxia in PDT. To demonstrate this, c(RGDfK)‐functionalized PLGA nanoparticles loaded with photosensitizer methylene blue (MB) and catalase in the aqueous core, and black hole quencher‐3 (BHQ‐3) in the polymer shell, were prepared. In the normal condition, BHQ‐3 effectively quenched the ^1^O_2_ production of MB and mitigated the adverse off‐target phototoxicity of nanoparticles until it reached the target cell. After selective uptake of the nanoparticles by α_v_β_3_ integrin‐rich tumor cells, H_2_O_2_ permeated into the core and was catalyzed by catalase to generate molecular O_2_, leading to the dissociation of the nanoparticles and release of MB (Figure 10B). The free de‐quenched MB under light irradiation induced the generation of ^1^O_2_, which eventually destroyed the cancer cells. Overall, target specificity and H_2_O_2_‐controlled and continuous O_2_‐generating capability of these nanoparticles might be highly promising to improve PDT efficacy in hypoxic tumor.

## 3 ROS‐Triggered Drug Release from Hydrogels

Hydrogels are three dimensional polymeric networks which generally have the capability to absorb large amounts of water or biological fluids.^61^, ^62^ These structures are mostly formed through crosslinking of hydrophilic polymer chains initiated by ionic, H‐bonding, hydrophobic interactions or chemical crosslinking. Because of their hydrophilicity and ability to mimic the extracellular matrix, hydrogels have become attractive biomaterials for the sustained and local delivery of therapeutic agents as well as supporting materials for cells during tissue regeneration. Among various hydrogels, injectable ones that can be formed in situ after being injected into the body offer high benefits over conventional preformed hydrogels because they can be administered via simple and minimally invasive procedure into the locations that are hardly accessible via surgery.^63^ Nevertheless, to harness the full potential of injectable hydrogels as a safe and effective drug delivery platform, it is also essential to impart specific drug releasing mechanism and in vivo biodegradability into them. The stimuli‐responsive moieties have been widely employed to develop intelligent or smart hydrogels as they can change the hydrophobic/hydrophilic balance, entanglements of polymers and degree of crosslinking which are important in maintaining the structure of hydrogel.^64^ Since various ROS‐responsive units can cause hydrophobic to hydrophilic transition or polymer chain scission by degradation of the linkages under oxidative environments, the incorporation of these ROS‐responsive moieties into the hydrogel structures is expected to improve their resulting drug release characteristics. Therefore, in this section, we will discuss drug delivery hydrogels which undergo solubility switch or cleavage under high level of ROS.

### 3.1 Hydrogels with Solubility Switch Units

**Figure 11:**
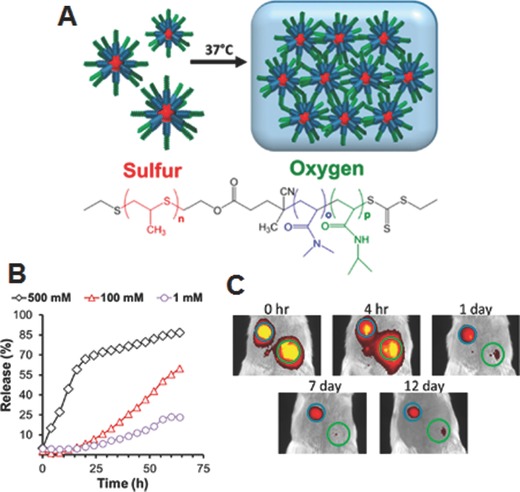
A) Chemical structure and illustration showing micelle gelation of PPS‐*b*‐PDMA‐*b*‐PNIPAM and ROS‐triggered release, B) H_2_O_2_‐concentration dependent Nile red release, and C) time‐dependent IVIS imaging of mice injected with diblock (green circle) and triblock copolymer (blue circle). Reproduced with permission.^65^ Copyright 2015, American Chemical Society.

Duvall and co‐workers demonstrated ROS‐triggered drug release from thermoresponsive hydrogels which were prepared using a ABC type triblock copolymer, poly [(propylenesulfide)‐*b*‐(*N,N*‐dimethylacrylamide)‐*b*‐(*N*‐isopropylacrylamide)] (PPS‐*b*‐PDMA‐*b* ‐PNIPAM) (**Figure** **11**A).^65^ At ambient temperature (25°C), this copolymer formed self‐assembled micellar nanostructures with a hydrophilic PNIPAM corona and a hydrophobic PPS inner‐core, which facilitated the loading of hydrophobic model drug Nile Red dye. When these assemblies were further heated to physiological temperature (37 °C), i.e., above low critical solution temperature (LCST) of PNIPAM, they transformed into stable hydrogels. As expected, upon exposing these hydrogels to H_2_O_2_, they showed a H_2_O_2_‐concentration dependent release of Nile red due to the oxidative solubility switch of PPS block (Figure 11B). When subcutaneously injected in male BALB/c mice, the sustained release of dye lasted over 14 days from the hydrogels, but the diblock copolymer composition without the PNIPAM block quickly cleared from the injection site within 1 day (Figure 11C), suggesting that thermo‐responsive gelation is critical for local drug retention. These results indicate that these hydrogels have potential as H_2_O_2_‐mediated on demand drug delivery system.

### 3.2 Hydrogels with Cleavable Units

Besides employing ROS‐mediated solubility switch mechanism, hydrogels integrated with ROS‐responsive cleavable units have also been reported for controlled drug release. This type of hydrogel undergoes polymer scission under oxidation conditions as a major mechanism to trigger drug release. The release rate can be effectively tuned by controlling the degree of incorporated cleavable units. Furthermore, it also guarantees complete degradation of hydrogel polymeric compositions into non‐toxic soluble molecules that can be excreted from the body, thereby evading unwanted immune response caused by prolong residence of the in situ formed gel.^63^

**Figure 12:**
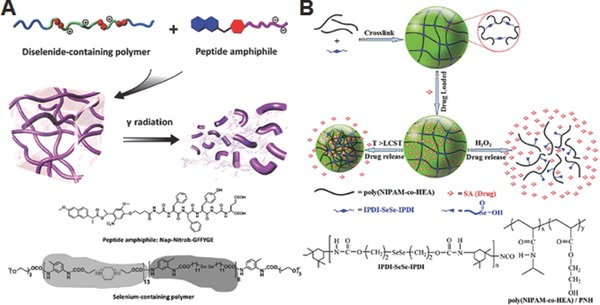
A) Schematic illustration of formation of supramolecular hydrogel from diselenide‐containing block copolymer and peptide amphiphile and γ‐radiation induced degradation. Reproduced with permission.^66^ B) Schematic illustration of formation of diselenide‐crosslinked hydrogels, and their oxidation and thermo‐responsive morphological change. Reproduced with permission.^67^ Copyright 2015, Royal Society of Chemistry.

Based on a ROS‐degradable diselenide‐containing block polymer and a peptide amphiphile as building blocks, Xu and co‐workers fabricated a supramolecular hydrogel which can respond to oxidative species produced by γ‐ray radiolysis of water (**Figure** **12**A).^66^ The hydrogel displayed a distinct gel‐sol transition for a 0.5 kGy dose of radiation, while a hydrogel analogue prepared with dynamic disulfide bonds using the same procedure failed to show any response even after exposed to a high dose of 5 kGy, indicating that gel–sol transition is proceed via cleavage of diselenide bond. As the peptide amphiphile is composed of a pain killer drug naproxen and a hexapeptide linked via photocleavable *o*‐nitrobenzyl ester group, irradiation of the gels with UV light also induced gel–sol transition and triggered the controlled release of naproxen. This result suggests that hydrogels made using diselenide‐containing polymer could serve as potential carrier to deliver drugs with radiation, especially for combination chemo and radiotherapy. By utilizing poly(NIPAM‐*co*‐2‐hydroxethyl acrylate) and ROS‐cleavable diselenide‐bearing crosslinking agents, Li et al. formulated oxidation and thermo‐responsive hydrogels for controlled delivery of salicyclic acid, a small molecular model drug (Figure 12B).^67^ These hydrogels were prepared by coupling reaction between the pendant –OH group of the copolymer and –NCO group of the crosslinker. The resulting gel networks quickly released the entrapped salicylic acid (over 90 wt% in 24 h at 25 °C) when treated with an oxidizing agent H_2_O_2_, which could be due to the dissociation of the bulk gels via cleavage of diselenide bond into selenic acid. Also, the hydrogels showed thermo‐induced drug release because its shrinkage promoted squeezing process above the LCST, but only 48 wt.% salicylic acid was release in 24 h at 40 °C. Although this study demonstrated preliminary oxidation‐induced burst or thermo‐induced sustained drug release from the crosslinked hydrogels, it did not provide any in vitro or in vivo drug releasing experiments mimicking physiologically relevant ROS conditions that are essential for real applications.

## 4 ROS‐Triggered Drug Release from Inorganic Nanoparticles

Mesoporous silica nanoparticles (MSNs) are one of the widely exploited inorganic nanoparticles due to their favorable structural properties, such as easy preparation and functionalization, tunable particle size and pore size, and excellent biocompatibility.^68^, ^69^, ^70^, ^71^, ^72^, ^73^ In addition, the pore‐surface biphasic structure of MSN allows not only to load various hydrophobic agents into their uniform mesoporous channel, but also to develop versatile drug delivery systems by endowing various functions including diagnosis, stimuli‐responsiveness and targeting ability to the delivery systems. In particular, MSNs are known to induce the ROS generation in several cell lines, which show their potential in the development of ROS‐responsive drug delivery systems.^74^, ^75^, ^76^, ^77^, ^78^, ^79^, ^80^ Therefore, in this section, we would like to discuss the MSN‐based ROS‐responsive drug delivery systems.

### 4.1 Linker‐Based System

**Figure 13:**
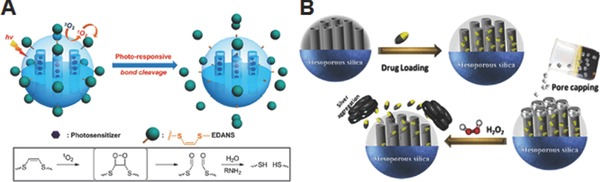
A) Schematic illustration of the mechanism of drug release by ^1^O_2_ produced from light‐activated photosensitizers. Reproduced with permission.^81^ Copyright 2013, Royal Society of Chemistry. B) Schematic illustration of preparation of H_2_O_2_ responsive drug delivery systems utilizing Ag NPs as a gatekeeper. Reproduced with permission.^82^ Copyright 2015, American Chemical Society.

One of the easiest ways to develop ROS‐responsive MSN drug delivery systems is to integrate ROS‐responsive linkers within the nanostructure. Accordingly, our groups developed photoresponsive drug releasing systems (Pc@AP‐E) based on MSNs by utilizing ^1^O_2_ sensitive linkers and ^1^O_2_‐generating photosensitizers (**Figure** **13**A).^81^ ZnPc which generates ^1^O_2_ in response to long‐wavelength light (*ca*. 600–700 nm) was loaded into the porous structure of MSNs via hydrophobic interactions, while 5‐[(2‐aminoethyl)amino]naphthalene‐1‐sulfonic acid, one of the model drugs, was decorated on the surfaces of MSNs via ^1^O_2_‐labile vinyldithioether linker. According to the design, light irradiation induced the ^1^O_2_ generation from the ZnPC in the pore of MSNs, which eventually facilitated the release of model drugs by cleaving the ^1^O_2_‐sensitive linkers between MSNs and model drugs. Although this initial stage research demonstrated the potential use of ^1^O_2_ as a trigger of on demand drug release systems using MSNs, their practical usefulness still needs to be verified in in vitro and in vivo systems.

### 4.2 Gatekeeper‐Based System

There are several reports on the development of MSN‐based ROS‐responsive drug delivery systems without utilizing ROS‐responsive small molecule linkers. Zhu and Du groups suggested a strategy utilizing metal nanoparticles as gatekeepers for ROS‐responsive drug delivery, which is based on the ROS‐responsive stability of metal nanoparticles. They hypothesized that the ROS‐responsive gatekeeper systems can be achieved if the metal nanoparticles that cap the pores of MSNs can be destabilized and removed from the MSNs by ROS. According to this logic, they employed the oxidative sensitive silver nanoparticles (Ag NPs) as gatekeepers to develop H_2_O_2_ sensitive drug delivery systems (Figure 13B).^82^ Ibuprofen drug was loaded in the nanochannels of MSNs and the pores were capped by citrate passivated Ag NPs with ultrasmall size (4–6 nm) to prevent the premature release of drug. When exposed to H_2_O_2_, the outer citrate‐stabilized surface of Ag NPs were etched and resultant destabilized Ag NPs immediately underwent the dissolution‐accompanied aggregation. That is, H_2_O_2_ exposure led to the uncapping process of mesoporous nanochannels and subsequent release of drug molecules. As with the case of using Ag NPs, they reported hydroxyl radical (·OH) responsive gatekeeper systems (ZnS@CPT@MSNs) utilizing zinc sulfide quantum dots (ZnS QDs) as gatekeepers in another study.^83^ Ultrasmall (3–4 nm in diameter) thiol stabilized ZnS QDs with free carboxylic acid groups was developed, followed by conjugating to amines groups in CPT‐loaded MSNs to cap the 3 nm wide pores of MSNs. Because highly reactive ·OH which can be generated via Fenton reaction between H_2_O_2_ and Fe^2+^, the most abundant transition‐metal in the body, induced destabilization of thiol groups on ZnS QDs, the ZnS QDs were aggregated and removed from the surface of MSNs. This process led to the release of CPT in controlled manner depending on the ·OH. Because the system could be operated by the biorelavent concentrations of H_2_O_2_ and Fe^2+^, the developed ZnS@CPT@MSNs exhibited high anticancer effects on BxPC‐3 cells. In addition, the reduced cytotoxicity of ZnS@CPT@MSNs in the presence of antioxidant vitamin C suggested that the intracellular ROS generation has an important role in the uncapping process and the release of CPT. Although the strategy utilizing metal nanoparticles as gatekeepers provides opportunity to avoid the use of ROS‐sensitive linkers whose synthesis and modification are generally difficult, the cytotoxicity and the renal clearance issue of metal nanoparticles must be verified in in vivo systems for practical applications.

## 5 ROS‐Activatable Prodrugs

In principle, prodrugs are inactive form of drug molecules that, following systemic administration, normally undergo enzymatic or chemical activation at the targeted site of the body to generate active parent drugs, which eventually elicit the desired pharmacological effects. The chief rationale behind the design of prodrugs is to improve the adverse properties of drugs such as poor solubility, lack of site specificity and inefficient cellular uptake. In fact, about 10% of globally marketed drugs are categorized as prodrugs, and this value is increasing as more drug developers utilize prodrug strategy to improve drug formulations.^84^, ^85^, ^86^ It is also interesting to note that approximately 20% of all new small molecular drugs approved between 2000 and 2008 were prodrugs.^85^ Indeed, many potent hydrophobic small molecule anticancer drugs have been successfully formulated as prodrugs to enable enhanced solubility and target specificity. Given the relatively high level of ROS in cancer cells, it is not surprising that ROS‐activatable prodrugs could be one of the promising strategies for tumor‐targeted therapy.^87^

**Figure 14:**
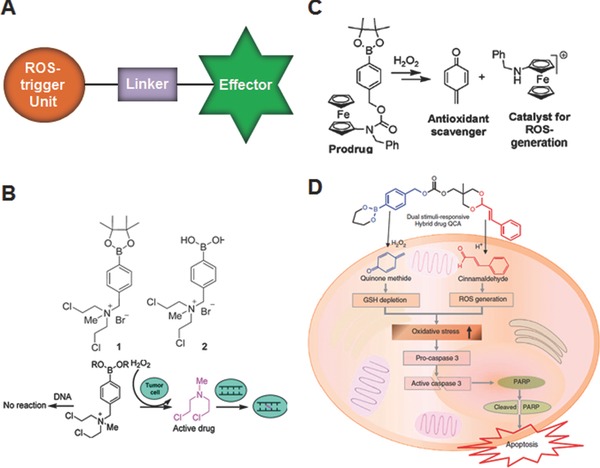
A) Cartoon representing general molecular design of ROS‐activatable prodrugs. B) Chemical structure of H_2_O_2_‐activatable DNA alkylating nitrogen mustard prodrugs and their mechanism of action. Reproduced with permission.^89^ Copyright 2011, American Chemical Society. C) Activation of ferrocene‐based prodrug in the presence of H_2_O_2_. Reproduced with permission.^92^ Copyright 2012, American Chemical Society. D) Intracellular activation of dual‐stimuli‐responsive prodrug. Reproduced with permission.^94^ Copyright 2015, Nature Publishing Group.

A modular design of ROS‐activatable prodrugs should encompass the following three functional domains: a ROS‐responsive trigger unit, an effector unit and a linker which joins both units together, as illustrated in **Figure** **14**A. In these types of activatable prodrugs, once the trigger unit undergoes ROS‐mediated metabolism in tumor cells, the linker should rapidly transmits the trigger signal and activates the effector to cause significant cytotoxic effect. The earliest examples of ROS‐activatable prodrugs have been designed mostly using arylboronic acid and their ester as a trigger unit.^88^ Because of their well‐defined and facile chemistry for chemical modification, DNA‐alkylating agents have been initially investigated as effectors for ROS‐activated prodrugs. For example, Peng et al. synthesized two H_2_O_2_‐activatable anticancer prodrugs (Figure 14B) by conjugating DNA‐alkylating nitrogen mustard with an arylboronate and arylboronic acid.^89^ Nitrogen mustards are one of the most useful DNA alkylating agents for cancer chemotherapy, but their applications are severely limited by lack of selectivity and toxicity. The as‐synthesized prodrugs exhibited low toxicity under normal conditions because of the positive charge developed on the nitrogen mustard, which requires for alkylation. On the contrary, in the presence of H_2_O_2_, the prodrugs rapidly released nitrogen mustard mechloroethamine and caused significant cytotoxicity by facilitating DNA alkylation and cross‐linking formation. *In vitro* studies of the prodrugs also demonstrated enhanced (60–90%) growth inhibition towards various cancer cells while the normal lymphocytes were less affected, indicating oxidative tumor specific activation of the nitrogen mustard is primary cause for effective cell killing. To enhance the cytotoxicity of H_2_O_2_‐activatable prodrugs, in a subsequent study, an attempt has also been made by these authors through conjugation of the trigger unit with more than one effector.^90^ The strategy utilizing arylboronic acid to develop H_2_O_2_‐activable prodrugs was also exploited for the targeted cancer therapy by reversibly modulating protein function.^91^ Xu groups modified lysine groups of cytotoxic protein ribonuclease A (RNase A) with 4‐nitrophenyl 4‐(4,4,5,5‐tetramethyl‐1,3,2‐dioxaborolan‐2‐yl)benzyl carbonate (NBC) to afford the RNase A‐NBC capable of modulating its cytotoxicity in tumor environment. According to the design, the high level of H_2_O_2_ triggers the boronic acid initiated 1,6‐elimination, which restores the activity of RNase A. The decreased isoelectric point (pI) of RNase A by boronic acid facilitated the electrostatic interactions with cationic lipid nanoparticles, which allowed the efficient intracellular delivery of the prodrug. Although the RNase A, RNase A‐NBC, vacant lipid nanoparticles exhibited negligible cytotoxicity in vitro, the RNase A‐NBC in lipid nanoparticles efficiently prohibited the growth of cancer cells. In particular, insignificant cytotoxicity was observed in noncancerous cells with low ROS levels, indicating that the developed H_2_O_2_‐responsive pro‐protein has a potential in developing tumor selective anticancer therapeutics.

Another promising approach to improve effectiveness and the target selectivity of ROS‐activatable prodrugs could be the strategy amplifying the intracellular ROS levels, because it not only increase the sensitivity of prodrug activation but also induce ROS‐mediated cancer cell death depending on the levels and duration of ROS exposure.^2^ Though cancer cells exhibit high oxidative stress with an increased production of ROS, they may not be able to survive above a certain threshold ROS levels. To ameliorate the adverse effects of heightened ROS, most cancer cells normally activate its ROS defense mechanism, often through the upregulation of GSH or other antioxidative molecules. Therefore, increasing the intracellular ROS by suppressing cellular antioxidant capacity and/or delivering exogenous ROS‐generating agents could be a potentially useful strategy for selective killing of malignant cancer cells. Based on this logical premise, to simultaneously inhibit the antitoxidative cellular system and induce generation of ROS, Mokhir group developed a H_2_O_2_‐activatable prodrug by conjugating an aminoferrocene derivative to a pinacol boronate ester via a carbamate linker (Figure 14C).^92^ In cancer cells with elevated ROS, this prodrug is able to generate an antioxidant QM and an aminoferrocene. The former one alkylates GSH, while the later one induces catalytic generation of hydroxyl radicals. Thus, this dual effect may cause a strong oxidative stress in cells, thereby leading to cell death. When tested against human promyelocytic leukemia cells (HL‐60) and human glioblastoma‐astrocytoma (U373), this prodrug showed potent cytotoxic effects with IC_50_ values of 9 μm and 25 μm, respectively, which are largely attributed to their site‐specific activation and expected synergistic action of the released products. The non‐malignant cells such as fibroblasts were less affected by the prodrugs under practical conditions. However, at high concentrations (10 μm) and prolonged incubation times (72 h), the prodrugs can also be activated in normal cells which are known to exhibit low levels of ROS, indicating optimal dosage and time course is critical for future applications.^93^ As a next step in this line of research, more recently, Lee and co‐workers have synthesized an interesting dual stimuli (ROS and pH)‐responsive prodrug that can deliver QM and ROS‐generating cinnamaldehyde (Figure 14D),^94^ which is a major component of cinnamon that have been widely used as food additive and considered to be safe. It has been reported that CA can induce apoptosis through ROS generation primarily in the mitochondria and inhibit growth of human cancer cells.^95^ As mentioned above, since tumor microenvironment and cell organelles exhibit acidic characteristic, the additional pH‐sensitive acetal linkage of the prodrugs may ensures effective release of the parent active agents for enhanced therapy. Based on the in vitro and in vivo studies performed using metastatic prostate cancer cells DU145, which are known to produce a large amount of ROS, they demonstrated this prodrug could produce synergistic anticancer effect by suppressing antioxidant system and generating ROS. Nonetheless, further detailed studies on pharmacokinetics and pharmacodynamics should be warranted to maximize its therapeutic potential.

As discussed in above sections, the integration of photosensitizer into the ROS‐responsive drug delivery systems facilitates the control of drug release on demand. The same rational was adopted by You groups to develop NIR‐responsive ROS‐activable prodrugs.^96^ The prodrug is comprised of a phorphyrin derivative, a ^1^O_2_‐responsive aminoarylate linker and a combretastatin A‐4, a natural anticancer drug inhibiting tubulin polymerization. When NIR is irradiated to the prodrug, the phorphyrin generates the ^1^O_2_. This ^1^O_2_ induces the cleavage of the aminoarylate linker and subsequent release of active combretastatin A‐4 drug. Notably, the developed drug not only showed the NIR‐dependent anticancer effects in vitro, but also exhibited high therapeutic effects in vivo.

**Figure 15:**
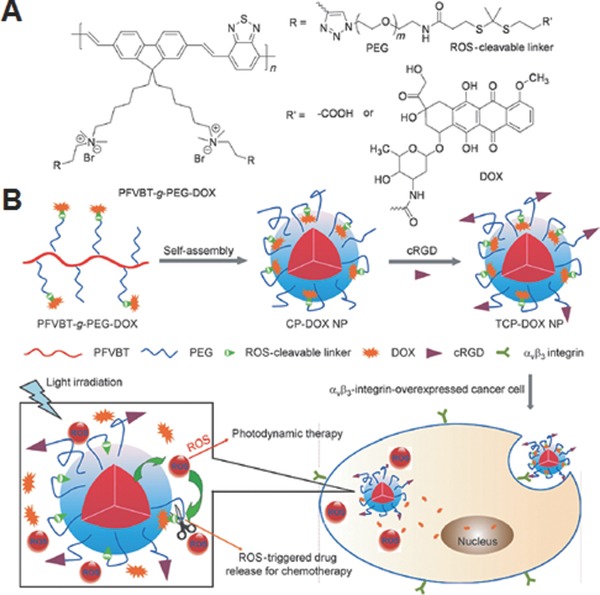
A) Chemical structure of ROS‐activatable polyprodrug, and B) preparation of self‐assembled nanoparticles using the polyprodrug and their light‐controlled ROS‐triggered drug release for combination chemo‐photodynamic therapy. Reproduced with permission.^102^

In recent years, activatable polymeric prodrugs (polyprodrugs), conjugation of active agents to polymers via biodegradable spacers, have received enormous attention because of their merits such as improved water solubility, enhanced in vivo stability, prolonged half‐life in blood, and specific tumor‐targeting characteristic via active or passive targeting mechanism (EPR effect).^97^, ^98^ Further, it also provides an excellent opportunity to integrate imaging and therapeutic capability into a single platform, referred as theranostics which is emerging as a promising strategy in personalized medicine because, in addition to therapy, it also facilitates monitoring real‐time localization of drugs and assess in vivo therapeutic efficacy following treatment.^99^, ^100^, ^101^ A representative example of ROS‐activatable polyprodrug has been provided by Yuan et al., who chemically conjugated DOX molecules to a conjugated polyelectrolyte via ROS‐cleavable dithioketal linkers (**Figure** **15**A).^102^ To impart solubility and targetability, they also conjugated hydrophilic PEG and cyclic ariginine‐glycine‐aspartic acid tripeptide (cRGD) which target cancer cells overexpressed with α_v_β_3_ integrins respectively to the polyelectrolyte backbone. The conjugated polyelectrolyte not only exhibits bright fluorescence for cellular imaging but also acts as a photosensitizer to generate in situ ROS for triggered on demand DOX release and PDT under appropriate irradiation of light. By simple dialysis, the polyprodrug conjugates were formed into self‐assembled nanoparticles (Figure 15B). Because of the combination of cRGD‐mediated targeting and white light‐controlled ROS‐triggered DOX‐mediated chemotherapy and PDT, these self‐assembled nanoparticles showed enhanced in vitro therapeutic activity on integrins α_v_β_3_ overexpressed MDA‐MBA‐231 cells. In spite of these favorable results, it is also important to highlight here that the use of biomedically benign and high tissue penetrable NIR light rather than white light is indispensable to realize the full potential of this system for clinical settings.

## 6 Conclusion and Perspectives

The intracellular ROS levels have been arisen as an important indicator for several diseases. Therefore, developing drug delivery systems which can respond to physiological relevant concentration of ROS have been significantly highlighted as a promising strategy in the field of drug delivery. Herein, we summarized and discussed the overall ROS‐responsive drug delivery systems from prodrugs and nano‐sized materials to macroscopic hydrogel. The successful development of ROS‐responsive drug delivery systems mostly depends on the availability of efficient ROS‐responsive materials. The diverse ROS‐responsive linkers discussed in this article trigger drug release from the carriers either by degradation or solubility switch (hydrophobic to hydrophilic) mechanism in response to ROS. The type of linker, sensitivity and its placement in the carrier system greatly influence the kinetics of the drug release. Each linker has its own advantages and limitations. For example, in spite of the high sensitivity of polymer containing organochalcogen (Se or Te)‐based linkers, their cytotoxicity is a crucial factor that needs to be evaluated. Similarly, the generation of harmful QM upon degradation of arylboronic ester linkers remains as a significant concern. Thus, it is highly important to ensure that the degraded linkers are still biocompatible until they are completely cleared from the body as non‐toxic fragments.

Although numerous ROS‐responsive drug delivery systems have been developed for biomedical applications, there are several challenges to be addressed for practical applications. First, the safety of the materials in addition to the linkers needs to be considered primarily. The materials employed as the building block of carriers should be highly biocompatible, otherwise they may lead to unwanted inflammatory responses and thereby elevates ROS level, which could inadvertently affect the programmed drug releasing characteristics. Second, for the systemically administered nanocarriers, the linkers incorporated in the carrier should be stable during circulation and at the normal cells with very low ROS levels, but effectively release the active agents after reaching at the pathological sites with increased ROS levels. Third, since there are many variations in patients, disease conditions and the level of expression of ROS, the selection of linkers and the carrier design need to be intensively considered for individual applications. Because of the complexity of the biological system, integrating other stimuli‐responsive moieties could be highly promising, and they could allow us to improve the therapeutic outcome by precise control of the carrier degradation and drug release rate. It is important to note that majority of ROS‐responsive drug delivery systems have not been evaluated in vivo. The in vitro results do not always necessarily guarantee the in vivo efficacy. Furthermore, the selection of real oxidative environment for in vivo evaluation also challenging, due to the rapid and dynamic changes in the ROS levels. Thus, efforts in integration of ROS‐responsive drug delivery systems with the diagnostic agents, referred as theranostic systems, may also help to advance these systems for more precise drug delivery. Also, the low intrinsic ROS level in the tumor might also be disadvantage for real in vivo application of ROS‐responsive drug delivery system. Therefore, new studies that employ ROS‐generating agents to increase tumoral ROS levels need to be explored. The ever‐continued endeavor in circumventing aformentioned challenges is expected to allow to achieve the practical therapeutic applications of ROS‐responsive drug delivery systems.
